# Rapid assessment of infrastructure of primary health care facilities – a relevant instrument for health care systems management

**DOI:** 10.1186/s12913-015-0838-8

**Published:** 2015-05-01

**Authors:** Stefan Scholz, Baltazar Ngoli, Steffen Flessa

**Affiliations:** University of Greifswald, Department of Health Care Management, Greifswald, Germany; GIZ Office Tanzania, Tanzanian German Programme to Support Health, Dar es Salaam, Tanzania

**Keywords:** Health care facility infrastructure, Rapid assessment tool, Tanzania, Health care facility management, Maintenance

## Abstract

**Background:**

Health care infrastructure constitutes a major component of the structural quality of a health system. Infrastructural deficiencies of health services are reported in literature and research. A number of instruments exist for the assessment of infrastructure. However, no easy-to-use instruments to assess health facility infrastructure in developing countries are available. Present tools are not applicable for a rapid assessment by health facility staff. Therefore, health information systems lack data on facility infrastructure.

**Methods:**

A rapid assessment tool for the infrastructure of primary health care facilities was developed by the authors and pilot-tested in Tanzania. The tool measures the quality of all infrastructural components comprehensively and with high standardization. Ratings use a 2-1-0 scheme which is frequently used in Tanzanian health care services. Infrastructural indicators and indices are obtained from the assessment and serve for reporting and tracing of interventions. The tool was pilot-tested in Tanga Region (Tanzania).

**Results:**

The pilot test covered seven primary care facilities in the range between dispensary and district hospital. The assessment encompassed the facilities as entities as well as 42 facility buildings and 80 pieces of technical medical equipment. A full assessment of facility infrastructure was undertaken by health care professionals while the rapid assessment was performed by facility staff. Serious infrastructural deficiencies were revealed. The rapid assessment tool proved a reliable instrument of routine data collection by health facility staff.

**Conclusions:**

The authors recommend integrating the rapid assessment tool in the health information systems of developing countries. Health authorities in a decentralized health system are thus enabled to detect infrastructural deficiencies and trace the effects of interventions. The tool can lay the data foundation for district facility infrastructure management.

**Electronic supplementary material:**

The online version of this article (doi:10.1186/s12913-015-0838-8) contains supplementary material, which is available to authorized users.

## Background

Health services research and health care management have frequently focussed on the role of supplies and personnel for the quantity and quality of health care services [1]. It is obvious that qualified and motivated personnel are crucial for preventive and curative medical services, and vaccines as well as drugs are of utmost importance for the health of people. However, the role of health care facility infrastructure as a major component of a health care system must not be underestimated. For instance, the WHO Alliance for Health Policy and Systems Research defines six building blocks of health care systems, the infrastructure constituting one component of the building block “service delivery” [2].

The term ‘infrastructure’ is used in manifold ways to describe the structural elements of systems. In the context of a health care system and in reference to health care facilities, we defined “facility infrastructure” as the total of all physical, technical and organizational components or assets that are prerequisites for the delivery of health care services. It can be seen as a major component of the structural quality of a health care system [3,4]. Same applies to health care facilities, i.e., functionality, quality and extent of such components and assets determine the accessibility, availability, quality and acceptability of health care services as well as the working conditions of facility staff [5-10].

**Figure 1:**
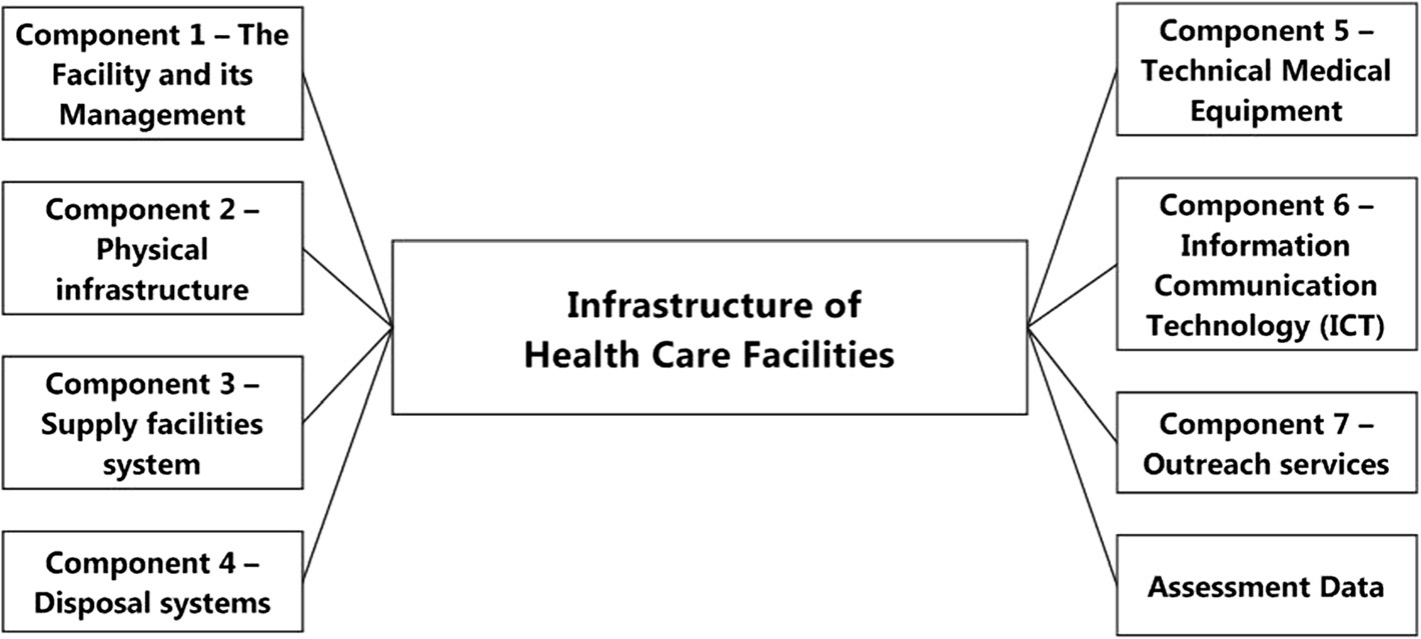
Health care facility infrastructure – major components. Figure 1 displays the seven major components that comprehensively describe all aspects of health care facility infrastructure. All these components have to be considered in the collection of data on facility infrastructure. Assessment specific data like date, name of data collector etc. complete the data collection.

Figure 1 displays the seven major components of the infrastructure of a health care facility: (1) the facility and its management, (2) the physical infrastructure, (3) the supply facility system, (4) the disposal system, (5) technical medical equipment, (6) information and communication technology, and (7) the outreach services. Most people associate with infrastructure only (2) and (5), but all components are prerequisites of a good structural quality.

Professional management is required to safeguard the functionality of all components. For instance, maintenance of infrastructure frequently constitutes a problem in resource poor countries [11]. It is frequently neglected due to lack of funds, availability of spare parts, poor training or little availability of maintenance personnel and a culture disregarding maintenance. Consequently, the condition of assets is often rather poor and contributes to the low structural quality of health care services [12]. This calls for a higher managerial awareness of infrastructure.

Although the relevance of health facility infrastructure for the health care quality is obvious, the literature on health care facility infrastructure is limited. In particular, there are no easy-to-use instruments to assess the quality of health care infrastructure. This article presents a rapid assessment tool which was developed for primary health care facilities in Tanzania. The main purpose is to demonstrate how this tool can provide an evidence-base for health policy decision-making by providing fast and reliable knowledge on the condition of health care infrastructure.

The next section provides an overview of existing health care infrastructure assessment instruments. Afterwards, we present the rapid assessment tool which was produced for Tanzania and an appraisal of this tool. The paper closes with a discussion on the integration of the tool in the health system.

### Assessment of health care infrastructure

A recent and rather comprehensive study on health care infrastructure was presented by Hsia et al. [13]. They evaluated six infrastructural key components of hospitals and health centres in African sub-Saharan countries under the aspect of access to surgical and emergency care. The study describes “…dramatic deficiencies in infrastructure […] in all countries studied” [13]. An older evaluation of the health care infrastructure of 16 Lutheran hospitals in Tanzania done by Flessa [14] came to the same results.

However, health policy decision-making cannot be based on snap-shot like research. Instead, routine data must be gathered within the existing Health Information Systems (HIS) [15-18]. Many developing countries currently implement the roll-out of a contemporary software-based HIS which includes epidemiological and demographic data. The majority of health data are gathered at the level of health care facilities. Data collection is performed by medical or administrative staff of the health care facilities in a monthly, quarterly or yearly routine. However, health information systems are reported to function insufficiently [16,19]. Furthermore, infrastructural components are only partly assessed even in recently developed tools like ‘Service Availability and Readiness Assessment’ (SARA) [20]. Assessments consider such components only to a minor extent in existing health information systems like MTUHA in Tanzania.

A reliable and sustainable health care facility infrastructure assessment tool must fulfil the following seven criteria: Firstly, a thorough collection of infrastructural data must cover all seven components of infrastructure (comprehensiveness). Secondly, the procedure must be a routine, i.e., data collection, assessment and recording must be simple enough that administrative or medical staff of (primary) health care facilities can perform it without professional support (easiness of use). Thirdly, data collection should not take too much time (rapidness) and, fourthly, it should fit into the existing HIS (adaptability). Fifthly, the tool and data collection process should be standardized so that the results are comparable (standardization). Sixthly, results have to constitute a reliable base for health policy decision-making including allocation decisions of central funds (usefulness). Finally, it must be easily adaptable to varying circumstances (flexibility).

These criteria which were derived from the desired function of the tool are in line with Rogers’ research on the diffusion of innovations [21]. Rogers emphasizes the importance of the perception of an innovation by the members of the respective social system. According to his research, the compatibility of an innovation is positively related to its rate of adoption, while complexity has the contrary effect. While various internal stakeholders of a health system [14] are involved in the use of an innovative assessment tool on facility infrastructure, the authors’ reflection of compatibility and complexity criteria primarily focussed on the first-line users i.e. the health care facility staff.

A number of assessment tools are available in developing countries that consider facility infrastructure, but they seem to fall short to fulfil the criteria stated above. Prominent examples for such tools are the ‘Service Availability and Readiness Assessment’ (SARA), the ‘Health Center Assessment Handbook’ and the methods of Kielmann et al. and of Halbwachs:

1. SARA, first published by WHO in 2012 [22], represents a very profound survey of manifold aspects of health care services. The tool is designed to be applied regularly as a systematic survey of facility service delivery using a standard questionnaire for the assessment [23]. It considers a number of aspects of infrastructure. It produces infrastructural indicators that describe aspects of service availability and service readiness (e.g. supply facilities, medical equipment and waste management). WHO published reports about assessments that used the SARA tool in Sierra Leone (2011) and Zambia (2010) [24]. The reports show the potential of SARA to display assessment results in a very distinguished way (e.g. general service readiness, differentiated as per facility type and in categories like basic amenities, basic equipment, and laboratory means etc.). This kind of data analysis and presentation of results is supportive for health policy decisions regarding interventions into the improvement of specific health services. However, this tool will usually require external professional staff to perform it [25]. Thus, it cannot be used as routine instrument for HIS in many developing countries.
2. The ‘Health Center Assessment Handbook’ of the Ethiopian Federal Ministry of Health, Planning and Programming Department and USAID [26] thoroughly describes a tool for a very detailed assessment of all facets of facility infrastructure. This method requires more professional human resources than all other tools.
3. Kielmann et al. [27] developed a comprehensive tool focusing on health needs, services and health care systems at district level. Among other aspects, the assessment provides a physical inventory of health facilities. Various components of physical infrastructure are enquired, but the tool does not cover all aspects of facility infrastructure (failing comprehensiveness). The advantage of this method is that it can be used by health care professionals without engineers.
4. Halbwachs [28] focuses on the appraisal of management of physical infrastructure of health services. His assessment tool can be applied at national as well as district and at facility level and encompasses “…a semi-quantitative and quick method of appraising the management of physical assets in health care”. The tool uses elements of the protocols for data collection developed by Kielmann et al. [27]. It is more detailed than the latter one, but it requires professional data collectors.

Consequently, none of these tools meets the above described requirements of rapid assessment and comprehensiveness. Tools differ in terms of extent of assessment, consideration of infrastructural data categories and use of ratings. All available tools presuppose professional expertise to a major extent. Only the ‘Health Center Assessment Handbook’ considers all components of facility infrastructure. All tools are more time-consuming than acceptable for a rapid assessment and require human or financial resources that exceed the possibilities of routine data collection in a HIS. There is no doubt that these tools are highly appropriate for scientific work or snap-shot like assessments for particular interventions (e.g. a major renovation program financed by development aid), but they cannot be applied for regular assessment within the HIS routine. In our opinion, even SARA which is supposed to be a practical tool for routine falls short of user friendliness and easiness to use.

To our knowledge no assessment tool is available that meets the requirements of rapid facility infrastructure assessment sufficiently. This results in insufficient quality of infrastructural data. Although a yearly professional assessment of facility infrastructure is desirable because it ensures better data quality the authors assumed that this will not be an option for the yearly data collection routine in resource-poor developing countries due to limited financial resources. Consequently, the object of our research was the development of a rapid assessment tool for health care facility infrastructure that can be applied by in-charge staff members of primary health care facilities. The tool shall contribute to the strengthening of primary health care services.

The rapid assessment tool was tested in a health district in Tanzania. At the same time, a civil engineer and a biomedical engineer performed a professional assessment. It included all data queries of the rapid assessment. The results of rapid and full assessment were compared.

The rapid assessment tool will be presented in the next section.

### Rapid assessment tool for primary health care facility infrastructure

In this section we describe the rapid assessment tool with its dimensions of data structure, rating schemes, indicators and indices. Afterwards the scope of a pilot test of the tool is outlined.

#### Data structure of rapid assessment tool

**Figure 2:**
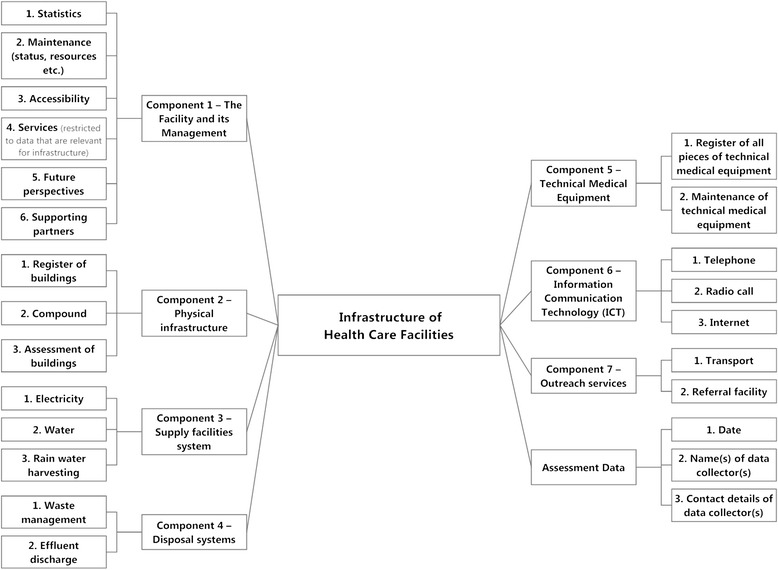
Aspects of facility infrastructure assessment. Figure 2 shows details of facility infrastructure assessment which describe aspects under the seven major components (compare figure 1).

The assessment comprises data that are collected for the facility as an entity, for specific buildings (building-wise) and for technical medical equipment (asset-wise). Types of data queries are “information” (e.g. date, time or name), “predefined text” (e.g. for rating the condition of roofing material) and “predefined ratings” of condition and of availability or reliability. Figure 2 shows the components of health care facility infrastructure for which data are collected.

**Figure 3:**
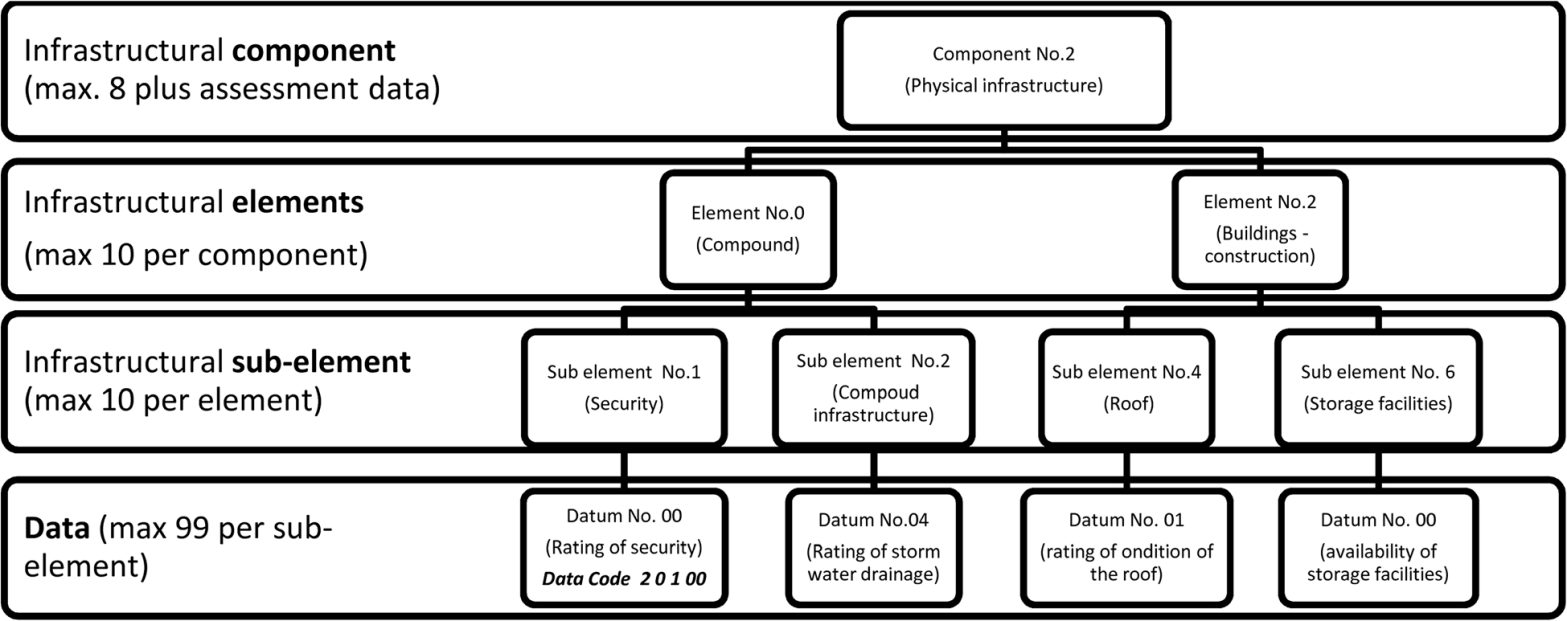
Data structure of rapid assessment tool – example. The data structure of the rapid assessment tool is explained in this figure, displaying an excerpt of infrastructural component number 2 (physical infrastructure), with the elements number 0 (compound) and 2 (buildings) and a selection of related sub-elements and data queries. The data code number 20100 is exemplarily indicated bottom left for ‘rating of security of compound’.

Data are organized in a hierarchy of 4 levels. Figure 3 depicts this exemplarily for a part of infrastructural component 2 (Physical infrastructure).

Data queries are numbered using five digits (e.g. “20100” stands for “rating of security of compound”, see data query bottom left in Figure 3).

The data structure is flexible for adaption to local circumstances. While a set of standard data queries must remain unchanged, specific locally required data queries can be added. However, any adaption to specific local circumstances reduces comparability of data in a wider context.

A maximum extent of standardization in the form of predefined answers and simple and consistent rating schemes grants the required easiness of use and the comparability of results. Thus, the adaption into a software-based HIS is facilitated.

The current version of the rapid assessment tool comprises a total of 101 data queries in 7 infrastructural components as shown in Table 1.

**Table 1:** **Rapid assessment tool – components, elements and number of data queries**

**Number**	**Infrastructural component (number of data queries)**	**Infrastructural elements in component (number of data queries)**	**Focus / Remarks**
1	The facility and its management (30)	Statistics (18)	The facility as an entity
		Maintenance (1)	
		Accessibility (1)	
		Information management (1)	
		Services (9)	
2	Physical infrastructure (26)	Compound (2)	Facility (entity)
		Buildings - basic data (5)	Buildings are
		Buildings – construction (7)	assessed discretely
		Buildings – interior works (6)	
		Buildings – installations (6)	
3	Supply facilities systems (16)	Electrical supply (5)	Facility (entity)
		Water supply (5)	
		Rain water harvesting (6)	
4	Disposal systems (14)	Waste (9)	Facility (entity)
		Effluent discharge (5)	
5	Technical medical equipment (7)	Asset location data (1)	Assets are assessed discretely
		Asset statistical data (2)	
		Asset functionality (2)	
		Maintenance resources (2)	
6	Information Communication Technology (4)	Telephone (2)	Facility (entity)
		Internet (2)	
7	Outreach services (4)	Transport (2)	Facility (entity)
		Referral (2)	

Table 1 refers to the seven major infrastructural components (compare Figure 1) and describes all infrastructural elements that are considered under rapid assessment, together with the related number of data queries.

An inventory of facility buildings needs to be compiled prior to the first rapid assessment of facility infrastructure. Same applies to technical medical equipment. Both inventories should be synchronized with standard lists (if available) prior to the assessment. If such standards are not defined on a national level, standard lists (as per facility level) can be developed by applying internationally accepted instruments, such as SARA [22] or Standards-Based Management and Recognition (SBM-R) of JHPIEGO [10,29].

#### Rating schemes

A number of data elements are assessed by using ratings. With reference to Halbwachs [28] ‘conditions’ are rated using the unified scheme ‘2-1-0’. The implications of ratings are:

- 2: very good or good condition; therefore no need of action;
- 1: minor problems; therefore need of action, but not immediate;
- 0: major problems or hazards; therefore need of immediate action.

These implications need to be explicitly explained to data collectors.

Explanatory text is added where necessary in order to adopt the wording of the ‘2-1-0’ scheme to the specific data requirements of the question (e.g. for ‘roof leakages’: ‘2 = no or minor leakages’, ‘1 = leakages require roof repair’, ‘0 = leakages cause major problems’).

Wherever applicable, data of the ‘information-type’ are transformed to ratings in the analysis of the assessment data according to defined standards. To give an example for such standards, dispensaries are expected to offer services 5 days per week (the datum ‘5 days’ is transformed to the rating ‘2’ for the calculation of the ‘accessibility indicator’).

#### Indicators, indices and standards

All ratings are grouped under infrastructural aspects and are used for the calculation of *indicators* that describe the different infrastructural aspects (e.g. ‘accessibility’). The calculation procedure takes into account that the requirements differ as per facility type.

**Figure 4:**
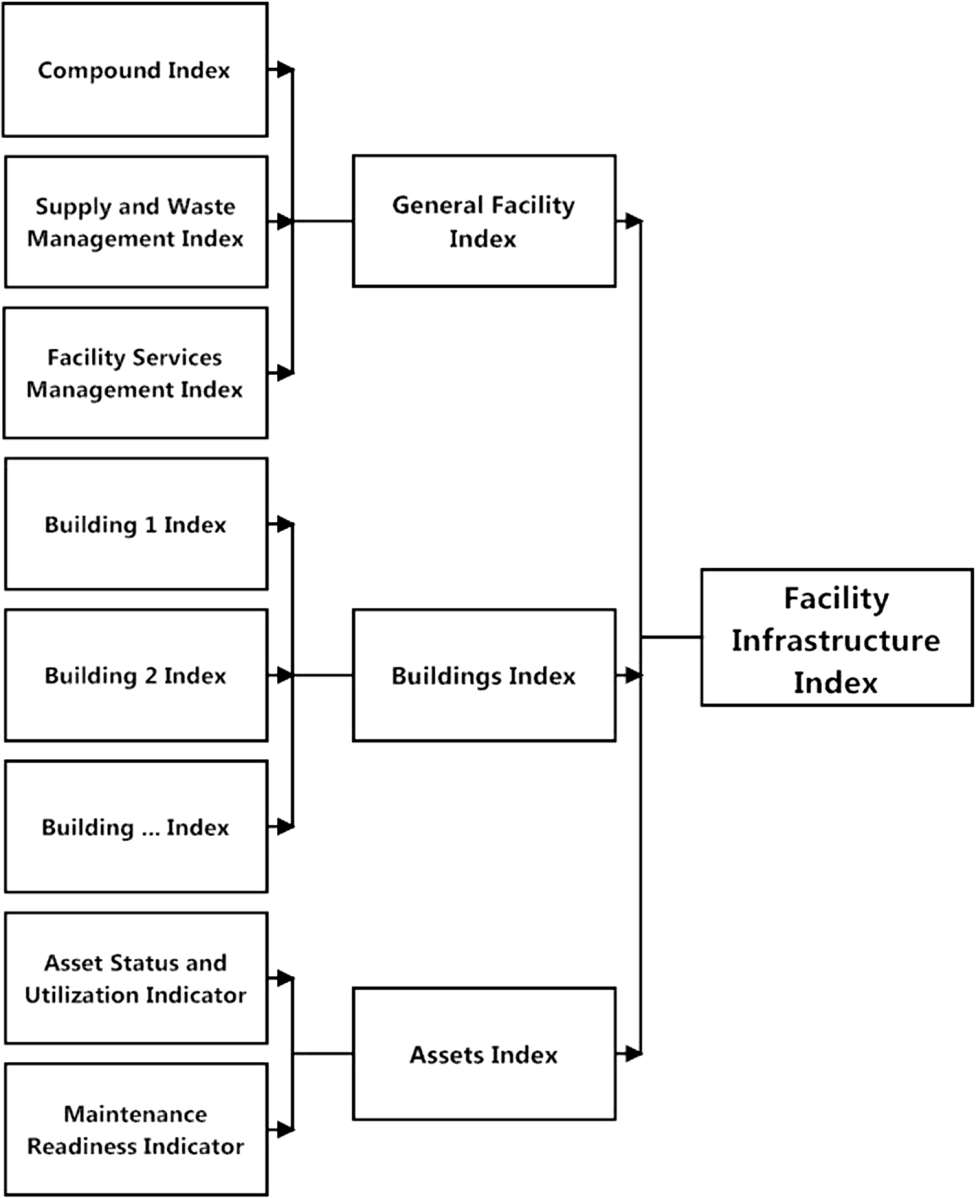
Indicators and indices of rapid assessment of health care facility infrastructure. Figure 4 depicts the systematic approach how infrastructural indicators and indices are calculated: data of the ‘rating-type’ are used for the calculation of indicators (for details, refer to Table 2). Indicators are combined under superior aspects (general facility aspects, buildings and medical technical equipment) forming infrastructural indices which finally enter into the ‘Facility Infrastructure Index’.

The infrastructural indicators are merged in *indices*. General facility indicators are combined in a ‘General Facility Index’. Building indices are calculated for all buildings separately and then combined in one ‘Buildings Index’. Asset indicators are combined in the calculation of the ‘Asset Index’. Finally the three major indices form a ‘Facility Infrastructure Index’. All calculations use arithmetic averages. Figure 4 depicts the systematic approach.

Infrastructural standards were not available for the field test and thus defined by the authors. Table 2 displays the infrastructural indicators obtainable from rapid assessment of facility infrastructure and the related number of data queries. *Scores* are calculated in accordance with the defined standards. For instance, the indicator “staff working conditions” achieves the maximum score of 100 if

- the facility offers a sufficient number of staff quarters AND
- staff quarters are in good condition and security is rated as good AND
- private washing facilities and toilets in a good and clean condition are available for staff members AND
- service and treatment rooms are equipped with separate hand washing facilities for staff members.

#### Field assessment in Tanga region

Questionnaires for full and rapid assessment were pilot-tested in a field assessment in Tanga Region (Tanzania) in August and September 2012. The full assessment was facilitated by Gesellschaft fuer Internationale Zusammenarbeit (GIZ) and executed by a team of professional engineers with a long working experience in the health care field. The questionnaires for rapid assessment were filled in by the In Charges of the facilities or by other responsible staff members. Data collectors were asked to skip questions in case the wording exceeded their English language skills (for an excerpt of the questionnaire, refer to Additional file 1).

**Table 2:** **Infrastructural indicators in rapid assessment**

**Object of rapid assessment**	**Infrastructural indicator**	**Number of data queries**
Facility	Accessibility	2
	Disposal safety	7
	ICT reliability	1
	Maintenance readiness	2
	Outreach possibilities	3
	Safety of compound	2
	Service readiness	4
	Supply capacity (water, rain water, electricity)	17
Buildings	Physical infrastructure condition	2
	Safety of rooms	6
	Staff working condition	9
	Storage safety	3
Technical medical equipment	Asset status and utilization	2

Table 2 lists all infrastructural indicators that are calculated from data collected by using the rapid assessment tool and the related number of data queries.

The scope of full assessment was a total number of 7 facilities with 42 buildings and 80 pieces of technical medical equipment. The rapid assessment covered 7 facilities and 42 buildings. Equipment was not the object of rapid assessment because the full assessment was done together with responsible staff members. If this had been followed instantly by a rapid assessment a lack of comparability of data would have been anticipated.

In the study the average time required for the assessment of dispensaries was 3 hours for full assessment / 1.5 for rapid assessment respectively 4.5/2.5 hours at health centres and 2 days /1 day at the district hospital.

## Results

The results have two dimensions. Firstly, we want to present briefly some results of the assessment itself. Secondly, we will present the results of the applicability of the rapid assessment tool, in particular in comparison to the full assessment undergone.

### Health care infrastructure in Tanga region

The gross result of the assessment was not really surprising, i.e., the assessment revealed infrastructural deficiencies. Infrastructural accessibility standards were achieved mainly, security standards partly. Service readiness was limited. Contrary to power supply, standards for water supply were not met at the majority of the facilities. The potential for rain water harvesting was only partly exploited.

Health care services were performed in buildings and rooms of insufficient infrastructural quality. Roof leakages and lack of plinth protection, including an insufficient rain water drainage, were major areas of concern. Facilities were not prepared for regular maintenance of technical medical equipment. They lacked functioning incinerators and separate disposal systems for infectious medical waste. The minority of dispensaries was equipped with placenta pits. All facilities reported problems with effluent discharge systems. Mobile phone reception was good at all facilities but only one facility provided office phones for communication. Web access was very limited.

The number of staff houses was insufficient. There was a lack of separate and clean facilities for hygiene of staff members. The need of maintenance of buildings and equipment was obvious but the facilities lacked capacities. This referred to qualified personnel, to space for workshops and to maintenance equipment.

### User-friendliness of the assessment tool

First of all, the interviews revealed a need to translate parts of the English questionnaires to Swahili in all dispensaries and in one health centre. Besides that, the reporting using indicators and indices in diagrams proved useful to point at specific infrastructural areas of concern. The field assessment showed that the tool’s data queries covered the components of facility infrastructure comprehensively. At none of the facilities and in both types of assessment, the data collectors pointed at elements of infrastructure that had not been considered in the development of the tool.

The pilot test showed that the building-specific approach of data collection was challenging to staff members but without alternative. Different buildings in the same facility showed very uneven condition of infrastructure. Without a separate assessment of every single building, infrastructural indicators and indices would give a false image of the actual infrastructural condition of a facility. Same applies to the asset-wise data collection on medical devices.

Using indicators and indices for verbal, numeric or graphic presentation of assessment results allows the easy highlighting of infrastructural components that require improvement. In relation with general health goals of a district, concretisation and prioritization of infrastructural measures is facilitated (for example, ‘improve rain water harvesting potential and its utilization at health centres in region X’ or ‘improve availability and functioning of basic sterilization equipment in dispensaries of Y district’).

The field assessment showed that staff members of all facilities were able to fill in the questionnaires for rapid assessment and to do this in a timely manner. The time required (between two hours and one day) was acceptable. Thus, the utilization of the tools – either in the form of questionnaires within the traditional reporting system or by using software – requires an adequate input of labour.

### Comparison of rapid and professional assessment

Data collected by facility staff using the rapid assessment tool (RAT) and by the engineers applying the full assessment tool (FAT) were compared in reference to general facility data, data on buildings and on supply and disposal facilities. Data entries (texts and ratings) of FAT and RAT were examined. Data were excluded from further appraisal if the RAT-data proved to be obviously wrong.

A total number of 284 ratings represented comparable data sets. Using the full professional assessment as reference, the detection of infrastructural deficiencies by use of the rapid assessment showed specificity of 82% and sensitivity of 71%.

The degree of urgency for interventions was rated higher in full assessment than in rapid assessment. This is assumed to have two reasons: firstly, the influence of the professional background of data collectors – an engineer’s expectations regarding the functioning and condition of infrastructure are likely to lead to a more critical rating; secondly, the habituation of staff members to deficiencies they have to cope with in their daily work is presumed to have a mitigating effect on the rating, while the professionals saw the infrastructure for the first time. It can be concluded that areas of infrastructure that require intervention are detected by both tools (rapid and full assessment), across all infrastructural components.

### Summary of field test

The survey showed that both assessment tools – full and rapid assessment –covered relevant aspects of the facilities’ infrastructure comprehensively. Standardization of data queries and the predefined and recurring rating schemes supported user friendliness and comparability of results. The field assessment revealed deficiencies of infrastructure and its management, resulting in an inefficient use of resources at the facilities.

In a nut-shell, the rapid assessment tool of health care facility infrastructure proved a reliable instrument of routine data collection by health facility staff that leads to results rather similar to the full assessment of professionals. That calls for its application as an integral instrument of the health information system.

## Discussion

Infrastructural data must become a natural component of the yearly routine data flow in a HIS as the melioration of facility infrastructure has the potential of improving health services [5,9,13] thus contributing to the final goal of better health of the population [17]. This unexploited potential should no longer be neglected.

The integration of data on health facility infrastructure into national health information systems in developing countries is overdue. Based on the findings of this case-study it is recommendable to integrate data on facility infrastructure in all health information systems of developing countries.

The rapid assessment tool explained in this article is ready to serve for this purpose. It is available in an excel spreadsheet version that was improved in evaluation of the field survey. The tool is transformable to software that is used for data collection in a HIS (e.g. by smart phones) and considers the capability of the facility staff acting as data collectors [15]. It provides a data structure that specifically measures all components and elements of facility infrastructure and is designed in accordance with the needs of a yearly data collection in existing health information systems.

The tool lays ground for data analysis in a structured reporting format and for the visualization of all relevant facets of facility infrastructure and possible malfunctions for reporting and management purposes. District health authorities are equipped with a tool which they own, which they apply and which they utilize for their decisions.

The regular rapid assessment of facility infrastructure has the potential to serve as an effective management tool for district facility infrastructure management.

### Limitations

To determine specificity and sensitivity of the rapid assessment test in this article, the full professional assessment was used as reference test. This assessment procedure requires further practice and evaluation to gain the status of a standard reference test.

The scope and design of the rapid assessment tool is at present limited to primary care facilities in the range between rural health posts and district hospitals. While the general assessment approach remains unchanged, the more complex infrastructure of referral facilities requires the definition of standards that specifically consider the hospitals’ functions.

The classification of medical devices in a rapid assessment requires a simple but effective scheme which has still to be developed. Existing systems like Global Medical Device Nomenclature [30] do not meet the simplicity requirements of rapid assessment.

The current lack of internationally agreed standards for health care facility infrastructure leads to a lack of comparability of results of infrastructure assessment above the national level.

## Conclusion

Applying WHO criteria [17], the collection and analysis of data on facility infrastructure by using the rapid assessment tool will enable health authorities (1) to improve the performance of a health care system by detecting and eliminating infrastructural deficiencies; this leads to better services, for example by assuring the availability and functioning of the required technical medical equipment; (2) to respond to threats by improving the reporting on facility infrastructure and related problems; (3) to improve health of the population which is, as above cited research proves, the consequence of improving accessibility, availability and quality of health services by providing good facility infrastructure. The instrument is ready and available for application.

## Additional files

Additional file 1:
**Rapid assessment tool, excerpt of questionnaire.** Format xlsx. This file shows an excerpt of the questionnaire for the rapid assessment of health facility infrastructure. All questions and data codes are given, together with the data types. Example for data type: ‘rating predefined’ for data code 40101 (functioning of incinerator) offers the following predefined answers: 2 = fully functioning OR 1 = minor problems OR 0 = major problems or not functioning.
